# Review of Possible Therapies in Treatment of Novichoks Poisoning and HAZMAT/CBRNE Approaches: State of the Art

**DOI:** 10.3390/jcm12062221

**Published:** 2023-03-13

**Authors:** Maciej Noga, Agata Michalska, Kamil Jurowski

**Affiliations:** 1 Department of Regulatory and Forensic Toxicology, Institute of Medical Expertises, ul. Aleksandrowska 67/93, 91-205 Łódź, Poland; 2 Institute of Medical Expertises, ul. Aleksandrowska 67/93, 91-205 Łódź, Poland; 3 Laboratory of Innovative Toxicological Research and Analyzes, Institute of Medical Studies, Medical College, Rzeszów University, Al. mjr. W. Kopisto 2a, 35-959 Rzeszów, Poland

**Keywords:** Novichoks, organophosphate, toxicology, HAZMAT, CBRNE, nerve agents

## Abstract

Novichoks-organophosphorus compounds belong to the nerve agents group, constituting the fourth generation of chemical warfare agents. The tremendous toxicity of Novichoks is assumed to be several times greater than that of VX, whereas no published experimental research supports this. They were surreptitiously created during the Cold War by the Soviet Union. Novichok’s toxic action mechanism consists of the inhibition of acetylcholinesterase activity. The review includes data on treating poisoning caused by OPs which could be used as guidelines for the therapy in case of Novichok exposure and HAZMAT/CBRNE approaches. Novichoks pose a severe threat due to their toxicity; however, there is insufficient information about the identity of A-series nerve agents. Filling in the missing data gaps will accelerate progress in improving protection against Novichoks and developing optimal therapy for treating poisoning casualties. Furthermore, introducing solutions to protect medical personnel in contact with a hazardous substance increases the chances of saving casualties of HAZMAT/CBRNE incidents.

## 1. Introduction

**Figure 1:**
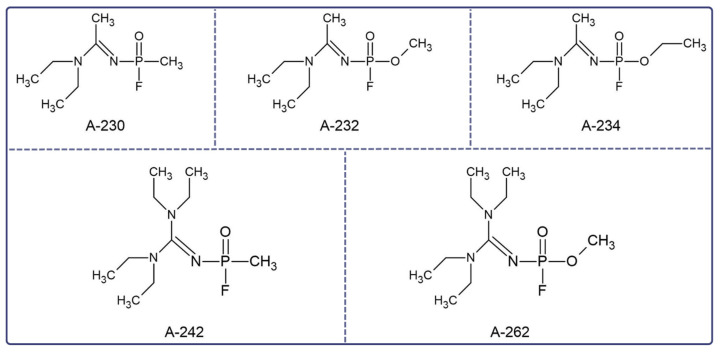
Novichoks’ structures developed at the State Scientific Research Institute for Organic Chemistry and Technology (GOSNIIOKhT) and revealed by Mirzayanov [2].

**Figure 2:**
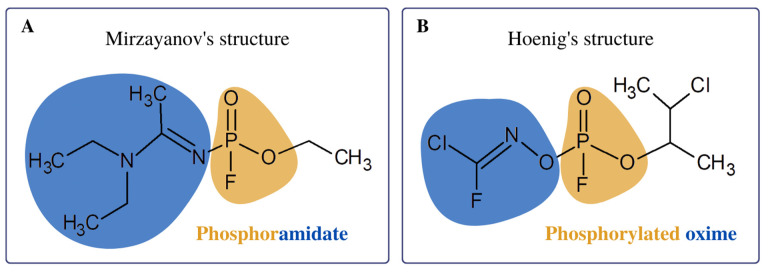
The chemical structures of Novichok A-234: (**A**) phosphoramidate and (**B**) phosphorylated oxime.

Novichoks (Russian: Hoвичóк, “newcomer”) belong to the group of chemical warfare agents with a paralytic and convulsive effect, referred to as A-series nerve agents (NAs) [1]. First, Vil S. Mirzajanov revealed information about the secret Soviet initiative to develop Novichok agents (Figure 1) in his book “State Secrets: An inside chronicle of the Russian chemical weapons program” [2]. It is postulated that Novichoks are organophosphates (OPs) containing a dihaloformaldoxime moiety [3]. Two chemical structures of Novichoks have been proposed: the first as phosphoramides (Figure 2A) published by Mirzayanov, and the second structures as phosphorylated oximes (Figure 2B), proposed by Hoenig [4] and Ellison [5].

**Figure 3:**
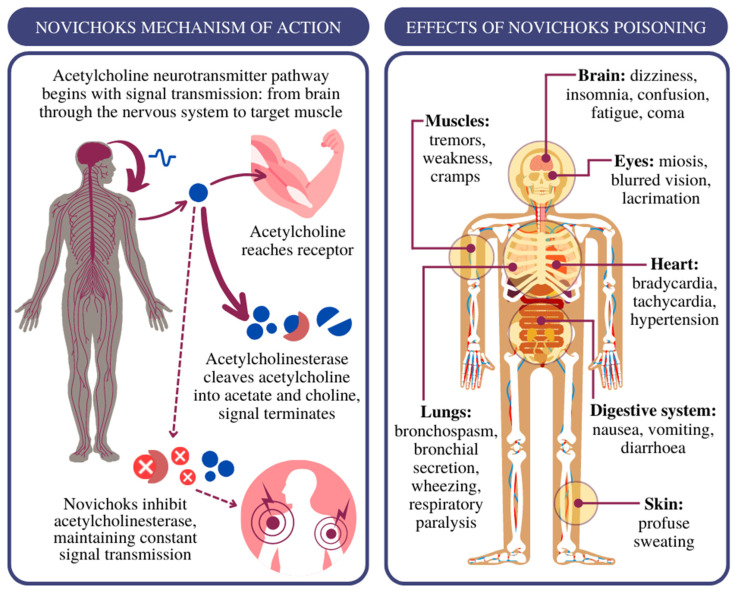
**Left** panel: the acetylcholine pathway in the cholinergic synapse and interaction with Novichok nerve agents; **Right** panel: effects of acute poisoning with nerve agents [7,11].

The primary assumption in the design of A-series compounds was the difficulty of their identification using standard equipment for chemical detection of the North Atlantic Treaty Organization (NATO) [6]. Novichok’s mechanism of action consists of irreversible binding to acetylcholinesterase (AChE) and inhibiting the hydrolysis of the neurotransmitter acetylcholine (ACh) to acetate and choline (Figure 3) [7]. At the molecular level, the formation of a covalent bond between AChE and Novichok not only slows down but completely terminates ACh hydrolysis. In tissue exposed to Novichok, ACh hydrolysis is slowed down because many AChE molecules will be covalently inhibited by Novichok. The mechanism of ACh and OP hydrolysis is the same, although ACh hydrolysis is much faster than OP hydrolysis, which can take hours or even days [8].

Overstimulation of cholinergic receptors caused by the accumulation of ACh in the synaptic cleft due to AChE inhibition leads to, depending on the route, dose and time of exposure, the manifestation of symptoms presented in Figure 3.

According to Mirzayanov, compound A-230 is 5–8 times more toxic than VX, while A-232 is 10 times more toxic than soman (GD) [2]. However, the calculations made by Carlsen assumed different data on the toxicity of Novichoks. The acute toxicity (LD_50_ value) of Novichok compounds are 5–75 times lower than compound VX [9]. According to the prediction data, the Novichoks’ hydrolysis rate is slower (several orders of magnitude) than the series G- and V- series compounds. Hydrolysis data would be valuable for defining the tenacity of Novichoks in the environment or the body [10].

Novichoks pose a constant and enormous danger due to their extreme toxicity. An example illustrating the threat of Novichok was the chemical attack on 4 March 2018 in Salisbury (United Kingdom). The compound of series A revealed a case of acute poisoning (of Sergei Skripal and his daughter Yulia), probably with the nerve agent A-234 [12]. A similar case occurred on 30 June 2018 in Amesbury (United Kingdom), where two British citizens were poisoned with the same compound as in Salisbury [13]. The third case of poisoning, due to which the application of an OP-NA from the Novichoks group was identified, which occurred on 20 August 2020 during a domestic flight in Russia. The formerly healthful 44-year-old man, about 10 min after departure, suddenly felt disoriented and began to sweat profusely, vomited, collapsed and then lost consciousness. Following the results of clinical and laboratory studies, complete inhibition of acetylcholinesterase in red blood cells was identified, thus confirming exposure to the cholinesterase inhibitor and no proof of obidoxime reactivation. The case of Navalny is the only published clinical study on the treatment of Novichok poisoning, which has proven the effectiveness of butyrylcholinesterase therapy supported by the administration of fresh frozen plasma [14].

The above examples confirmed the effects of acute poisoning with Novichok compounds, indicating their presence in public spaces. For this reason, it is a crucial task to treat poisoning with these highly harmful substances. Clinical treatment of exposure to the G- and V- series of NAs and their extrapolation to Novichok seem reasonable. In addition, appropriate procedure specifics related to the decontamination of hazardous materials (HAZMAT) or during chemical, biological, radiological, nuclear or explosive (CBRNE) incidents should be prepared. Given the global threat posed by Novichok, accurate and reliable data must still be provided to explain the unknowns.

## 2. Treatment of Novichok Exposure: Where to Start?

In general, treatment of poisoning caused by NAs can be divided into nonpharmacological and pharmacological treatment. Decontamination is one of the non-pharmacological methods. The aim is to limit further exposure to the nerve factor. Different approaches are used according to national standards [15]. Decontaminating the accident scene is essential to prevent bystanders’ poisoning. Vapors trapped in garments exposed to Novichok compounds can be released for up to 30 min [7]. Based on the similarity in the structure of the Novichok to other NAs, basic solutions might neutralize the A-series compounds. Increasing the pH of the solution could accelerate the reaction of Novichok hydrolysis [16]. The activation energy of hydrolysis for compound A-234 under basic conditions is lower compared to neutral conditions [17]. Theoretical calculations of the hydrolysis reactions of Novichoks confirm the greater effectiveness of decontamination under basic conditions. During decontamination, the dry bleach powder may hydrolyse Novichok compounds to hydrogen cyanide, hydrofluoric acid and hydrochloric acid. Therefore, it is recommended to avoid using these substances due to the formation of toxic metabolites [15]. Oxygen supply and resuscitation are non-pharmacological treatments in addition to decontamination [18].

The method of pharmacological treatment of NA poisoning, which could be applied to Novichoks poisoning as well, consists of the administration of (1) an anticholinergic agent (atropine), (2) an anticonvulsant agent (diazepam) and (3) the use of AChE reactivation agents (oxime) [19,20].

Immediately after poisoning with a Novichok group, clinicians should administer atropine, which will act as a competitive antagonist of central and peripheral muscarinic ACh receptors. Unfortunately, atropine is ineffective in neutralising the effects of overstimulation of neuromuscular nicotinic receptors overstimulation [21]. Atropine in doses of 2 mg to 6 mg with an interval of 5–10 min should be administered intravenously until bradycardia, bronchial bleeding and bronchospasm subside [7]. Atropine, in addition to intravenous administration, is also absorbed through the bronchial tree so that it can be administered by inhalation, for example, as an FDA-approved “Medical aerosolised nerve agent antidote” (MANAA) [22]. The impact of atropine has been shown to last from 3 to 5 h after 1–2 injections of 2 mg and 12–24 h after atroponisation [23]. A possible substitute for atropine, although tested in animal models, is the administration of the muscarinic acetylcholine receptor antagonist: diphenhydramine [24]. A drug called glycopyrrolate, in combination with atropine, was originally found to be suitable for controlling symptoms of OP poisoning and reducing central nervous toxicity [25]. Unfortunately, later studies contradicted these reports, claiming that the use of atropine alone causes fewer complications and results in lower mortality caused by organophosphorus compounds than the combination of atropine and glycopyrrolate [26,27]. On the contrary, atropine can be effectively replaced with glycopyrrolate in hypersensitivity; the recommended dose of the drug is 1 mg every 10–15 min until an antimuscarinic effect occurs [28]. Scopolamine may also serve as a substitute for atropine in hypersensitivity, used at a dose of 0.6–2 mg administered intramuscularly or intravenously. Scopolamine shows higher levels of CNS bioavailability compared to atropine [29]. In general, compounds that might block cholinergic effects should be detoxifying [30]. For example, benactizine, used as an antidepressant, is a fat-soluble anticholinergic drug, which allows it to penetrate the CNS more easily than atropine [31]. Moreover, it is additionally more efficient in preventing seizures than diazepam [32].

Diazepam prevents convulsions caused by Novichok poisoning, administered intravenously at 10–40 mg daily [33]. In addition to the anticonvulsant effect, diazepam has a specific effect on the cholinergic and GABAergic systems [34]. In animal models, diazepam has also been shown to reduce NA-induced brain damage [35]. Other benzodiazepines with effective anticonvulsant effects are lorazepam and midazolam. Because midazolam is more effective and faster than diazepam in stopping Novichok-induced seizures, it is recommended to replace the first with the second during urgent anticonvulsant treatment [36,37]. The recommended initial dose of lorazepam is 0.1 to 0.2 mg/kg (2–4 mg), and midazolam is 2.2 mg/kg [23]. Barbiturates and phenytoin have proven to be ineffective anticonvulsants in preventing seizures caused by NA, hence similar findings can be related to Novichoks [38]. Possible supportive therapy includes intravenous administration of a lipid emulsion in critically ill patients [39].

**Figure 4:**
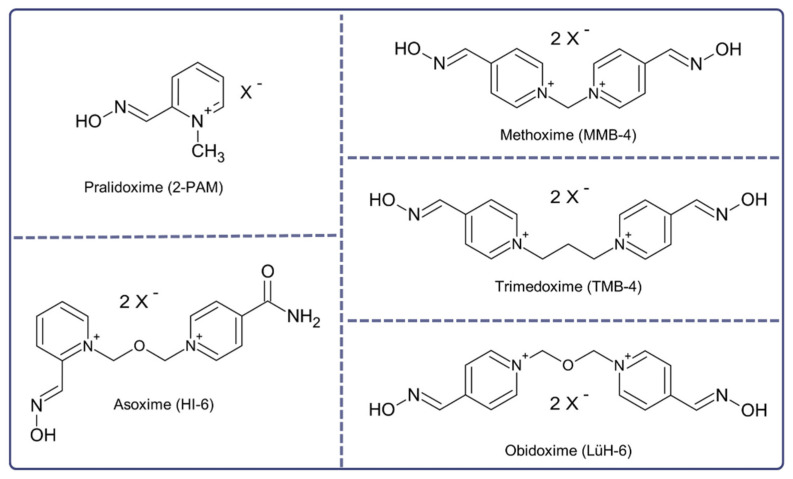
Chemical structures of oxime reactivators as an antidote against poisoning caused by nerve agents [42].

Oximes (Figure 4) were created to supplement atropine, which is ineffective against nicotinic effects and does not restore AChE activity inhibited by Novichoks. These nucleophiles reactivate the phosphylated cholinesterase enzyme by breaking the bond between the enzyme and Novichoks. Standard oximes approved for clinical and military use include 2-PAM, obidoxime, HI-6, and TMB-4, although their effectiveness is not universal [40]. The difficulty encountered by pyridinium oximes is passing through the blood–brain barrier (BBB). Because they are hydrophilic and have a positively charged nitrogen atom, only 1% to 10% of the plasma concentration of oximes is present in the brain. Therefore, the effect is limited mainly to the PNS [41].

Pralidoxime (2-PAM) should be administered intravenously in a dose of 1–2 g every 3 to 6 h or as a continuous infusion for a minimum of 24 h after the last intake of atropine [43]. Side effects in humans, such as blurred vision, double vision, transient dizziness and increased diastolic blood pressure, are minimal at therapeutic doses of 2-PAM and can depend on the rate of administration [44]. It is crucial to monitor liver enzyme levels regularly in patients who receive 2-PAM at doses of 1200 to 1800 mg by auto-injectors; usually, enzyme levels return to normal within two weeks [45]. The preferred route of administration of 2-PAM in hospitals is intravenous; however, there is also a route of intramuscular administration via an auto-injector with a dose of 600 mg/2 mL [38]. For casualties with severe central nervous and respiratory symptoms, three DuoDote auto-injection kits containing 600 mg of pralidoxime chloride are recommended, together with 2.1 mg of atropine [46]. Obidoxime is another oxime administered intravenously at a dose of 250 mg to reactivate AChE [47]. Due to hepatotoxicity, an initial dose of 500 mg and a daily dose of 750 mg/day of obidoxime are not recommended. Furthermore, liver function tests should be regularly monitored during therapy [34]. Obidoxime has been shown to be unable to reactivate acetylcholinesterase in red blood cells in a patient exposed to Novichoks. Furthermore, signs of liver damage with increased levels of transaminases and γ-glutamyltransferases have been partly attributed to obidoxime. This resulted in the early termination of obidoxime administration to the patient and recognition as an ineffective therapy [14]. In addition to the oximes mentioned above, asoxime chloride (HI-6) restores the activity of AChE inhibited by OP [21]. This double positive charged bis-pyridinium compound has been shown to be effective in the treatment of neural factors [48,49]. A bis-pyridinium oxime with superior therapeutic and pharmaceutical properties compared to both obidoxime and 2-PAM is a methoxime (MMB-4) [50]. An oxime with restricted use due to CNS and hepatotoxic side effects is thimodexime bromide (TMB-4) despite its effectiveness in reactivating AChE [51]. K203, a member of the new generation of oximes known as K-oximes, does not surpass the versatility of currently accessible oximes. On the contrary, its toxicity profile showed no perturbations compared to clinically used standards. K203 could be regarded as a backup or uncertain replacement for obidoxime and trimedoxime [52]. To our knowledge, the effectiveness of currently available oximes: 2-PAM, TMB-4, obidoxime and HI-6, in the reactivation of AChE inhibited by the Novichok A-242 surrogate, was evaluated. A combination of experimental and theoretical studies indicated that the most effective oxime for reactivating AChE inhibited by A-242 was trimedoxime (TMB), since they cannot adequately approach the substitute [53]. The newly synthesised phenoxyalkylpyridinium oximes managed to enter the brain and reverse the majority of NA-induced AChE inhibition. They were designed by adding an additional lipophilic chain to the pyridinium ring [54].

Another option in counteracting Ops as well as Novichock is the use of pretreatment like carbamate anticholinesterase, for example, pyridostigmine [55]. It is a reversible AChE inhibitor whose binding protects the enzyme from inhibition by NA [56]. The current pretreatment procedure is 30 mg every 8 h [55]. Pyridostigmine can be given orally (liquid or pill), intramuscularly or intravenously as injections to patients who cannot take oral drugs [57]. Pyridostigmine has been approved for military use by the FDA as a pretreatment to the GD [58]. The effectiveness of the prophylactic use of pyridostigmine depends on the quick use of atropine and pralidoxime (2-PAM) after exposure to a chemical warfare agent [55].

An alternative approach to poisoning treatment is the use of non-oxime compounds. Bispyridinium MB327 represents one of them, and the action is to reverse the neuromuscular blocking action of the OP compounds [59]. Animal studies have shown its protective efficacy against various NAs [60]. Unfortunately, there is no research on the effectiveness of MB327 on Novichoks, although the approach seems promising. Mannich phenol is a non-oxime that can reactivate an inhibited AChE enzyme, and 4-amino-2-[(diethylamino)methyl]phenol (ADOC) seems to be particularly interesting [61]. Based on the structure of Mannich phenols, more than a dozen non-oxime compounds capable of reactivating human AChE inhibited by NAs were synthesised [62,63]. At this point, we possess insufficient human studies on novel oximes, so currently, in the case of Novichok poisoning, commercially available oximes are still used.

Magnesium sulphate appears to be of note when treating patients with OP poisoning. This compound blocks calcium channels, which reduces the release of ACh. Moreover, it reduces excessive CNS stimulation resulting from activation of the N-methyl-D-aspartate receptor (NMDA) [64]. Intravenous administration of magnesium sulphate in a dose of 4 g on the first day shortens hospitalisation time and improves treatment results in patients with OP pesticide poisoning [65]. Furthermore, alkalisation of the blood with sodium bicarbonate and magnesium sulphate administration appears to be effective in recovering from moderate to severe poisoning. An infusion of 4 to 6 grammes of 20% MgSO_4_ solution within 24 h may decrease atropine needs, count of intubations and number of days in the ICU in OP toxicity [66,67]. The use of magnesium sulphate in Novichoks poisoning still needs further research.

Anti-glutamate and anti-NMDA drugs could also be interesting for OP-poisoning treatment. The excitatory amino acid glutamate plays an essential role in the continuation of OP-induced seizures through excessive activation of NMDA [68]. One of the anti-NMDA compounds approved for human use in neurotraumatology is gacyclidine. It has been shown in an animal model that gacyclidine administration can prevent neuropathology three weeks after exposure to OP, but there is no significant penetration into the CNS [22]. Gacyclidine provides optimal neuropathological protection when administered up to half an hour after poisoning with an organophosphorus compound [69]. Natural alkaloid huperzine A is an NMDA receptor antagonist and a reversible AChE inhibitor that can cross the BBB [70]. Huperzine A effectively protects peripheral and CNS AChE from OP compound poisoning [71]. Moreover, it prevents seizures and epilepticus after exposure by blocking NMDA-induced excitation [22]. Ketamine is another compound that can effectively stop epileptic seizures. This uncompetitive NMDA receptor antagonist is an appropriate epilepticus status prevention strategy after exposure [45]. In animal studies, ketamine and atropine have been shown to inhibit neutrophil infiltration and partially inhibit glial activity as significant neuroprotective effects [72].

The utilisation of antioxidants appears to be a reasonable approach to treating poisoning caused by organophosphorus compounds, which reduce total antioxidant capacity by generating nitric oxide and reactive oxygen radicals. Thus, they enhance the intensity of lipid peroxidation and increase the amount of reactive thiobarbituric substances. Vitamin E has been shown to have a therapeutic effect on OP-induced oxidative stress in rat erythrocytes [22,34]. Unfortunately, there is lack of information about application of vitamin E in Novichok’s poisoning.

A new modern antidote based on hexahistidine-tagged organophosphorus hydrolase (His6-OPH, EC 3.1.8.1) proved to be effective in detoxifying OP in vivo [73].

Bioscavangers are worth considering as an alternative treatment for Novichok poisoning. An example of such bioscavengers is human butyrylcholinesterase (HuBuChE), whose detoxification mechanism of OP compounds is based on stoichiometric binding one mole of organophosphate neutralised by one mole of enzyme [74]. BChE clears the OP from the bloodstream before interacting with the AChE, protecting the native AChE from inhibition of OP. Using bioscavengers, the side effects of current antidotes can be avoided, and the need for prompt administration of antidotes will be reduced [75]. The study on soman-poisoned macaques showed that an intramuscularly administered dose of 13.1 mg/kg was protective [73]. It is the basis for research on the effect of this bioscavenger in the case of exposure to Novichoks. Studies in animal models suggest that therapeutic concentrations of HuBChE in the blood can be maintained for at least four days after a single dose. First, it is safe for humans and does not show tissue toxicity [74]. BChE activity tests are widely available in routine clinical practise, primarily used as liver function tests. However, they are usually the only laboratory parameter that confirms the clinical diagnosis of organophosphate poisoning. The administration of BChE in the case of Navalny’s poisoning turned out to be the only effective treatment that saved the patient’s life [14]. Another bioscavenger is fetal bovine serum AChE (FBSAChE), which protects mice from multiple LD_50′_s of nerve agents [45]. Fresh frozen plasma (FFP) with albumin proved ineffective as a bioscavenger during a clinical trial in treating organophosphate-poisoned patients [76]. However, the data mentioned above are completely inconsistent with treating a human poisoned with Novichok, in which FFP was used with complete success. Administration of six units of FFP led to a marked increase in butyrylcholinesterase activity without a subsequent decrease, thus excluding the consumption of butyrylcholinesterase by an unbound inhibitory nerve agent in the bloodstream [14]. An interesting concept is the encapsulation of BChE, such an enzyme in a nanocarrier could more efficiently penetrate the BBB, which is associated with more effective treatment of acute neurotoxic effects of poisoning with organophosphorus compounds [77].

## 3. HAZMAT/CBRNE Approaches

There are two types of exposure to a hazardous material: HAZMAT (Hazardous Materials) and CBRNE (C—Chemical, B—Biological, R—Radiological, N—Nuclear, E—Explosives) [78]. The first type of event is unintentional and is randomly created, e.g., by an accident, breakdown or catastrophe [79]. On the other hand, the second type of event consists of the intentional release of hazardous materials into the environment, e.g., by terrorist or military activities [80]. In contact with a hazardous substance such as Novichok, the most crucial action to take in the first place is to neutralise the hazardous factor. Next, a pyrotechnic inspection of the place and event participants is carried out. The general procedure of emergency medical services in the event of exposure to a hazardous substance consists of the following steps [81]: preliminary identification of the threat, securing the incident scene, evacuating casualties from the danger zone, triage and preparation for decontamination and patient supervision during it, securing patients at a medical point and preparing for transport, carrying out transport, and medical support for rescue operations. Personnel requires correct protective equipment to implement the above action plan. Appropriate personal protective equipment (PPE) is necessary to protect paramedics and enable them to operate under hazardous environmental conditions. PPE consists of respiratory protection equipment and protective suits (prevention against skin contamination). The ideal solution for rescuers is universal protective clothing that ensures safety in various events and allows the implementation of rescue activities necessary to save lives [82]. OSHA (Occupational Safety and Health Administration) compliant contamination protective equipment classification is as follows:

- Level A, a gas-tight protective suit with a self-contained breathing apparatus structure, prevents the implementation of medical procedures;
- Level B, a protective suit with thin but solid fabrics and special protective coatings, is equipped with a self-contained breathing apparatus;
- Level C, a protective suit with a filter mask or escape hood with a wide visor and a powered air respirator;
- Level D, essential protective clothing (goggles and mask with a P3 filter), provides adequate protection in daily practise [81].

The system of dividing the area of operation into three zones has been specified to improve the organisation of rescue operations [83]: first, a hot zone where a hazardous substance is present and casualties are exposed to its effects. Entering the hot zone requires the use of PPE. Life-support activities in the hot zone include haemorrhage control, airway management and securing the patient during evacuation. Second, the warm zone initially lacks a hazardous substance. The first clean zone is where victims are evacuated. In this area, initial triage, cardiopulmonary resuscitation, protection of the patient’s basic vital functions and preparation for decontamination are performed. Third, the cold zone is free from hazardous substances. This is a safe area for paramedics who use essential PPE. This division of the area significantly facilitates the organisation of medical activities and the overall management of risk.

### 3.1. Personal Protection Equipment

Novichoks were designed as nerve agents that could not be stopped by the chemical protective gear available to NATO soldiers at that time [84]. Surprisingly, there is still a lack of relevant and appropriate review articles on HAZMAT/CBRNE approaches according to Novichok poisoning. Therefore, this is the first review of available data on this important and extraordinary topic [13,85,86].

First responders and medical personnel must ensure their own protection. Protection clothing and respiratory protection are the key to treating Novichok vapour or liquid exposure patients. Decontamination must occur outside the hospital, and many hospitals have a separate decontamination facility for self-tidy patients. HAZMAT personnel must establish a decontamination site for ambulatory and stretch cases. It should be noted that, since the first suspected Novichok poisoning (during the night on 4 March 2018), the attention has been focused on ensuring the safety not only of patients (decontaminated with thorough washing as part of our daily routine) but also of the staff who care for them. PPE has increased from standard precautions to different levels of protective clothing and equipment. Due to the new nature of Novichok and unknown physical properties, the necessary levels of protection were not initially well understood, and the recommended levels of PPE varied over time. When Novichok’s threats were better understood, the PPE required to care for patients was a single pair of disposable long sleeves, disposable long nylon gloves (to cover the sleeves), disposable surgical masks and eye protection for high-risk procedures. It is important to remember not only to treat patients but also to use PPE to handle potentially contaminated materials and equipment that should be appropriately contaminated. A lesson for any possible CBRNE incident is to start with high-level PPE and de-escalate only when the risk is clearer and more apparent. Fortunately, the agents involved seemed not volatile (as opposed to the 1995 Tokyo subway sarin attack, where the volatile agents threatened or affected first responders and health staff), and the level of contamination was limited but still significant. It should be emphasised that the strong persistence and hydrophobic nature of Novichoks, the removal of clothing from individuals and the decontamination before entering a screening area can prevent other patients and first responders from being exposed to the agent [87].

### 3.2. Supplies

The important issue is also supplies. The specific treatment regimen rapidly consumed available pharmaceutical countermeasures in the first accident mentioned. Interestingly, Salisbury District Hospital had a regional inventory of certain specific antidotes, so we immediately had sufficient pyridine for initial treatment (although we consumed a total supply of atropine in 24 h). The NHS blood transfusion service in the United Kingdom has a national reserve stock of pharmaceutical countermeasures for major events such as CBRN. The procurement also procured sufficient quantities of PPE while other teams coordinated the disposal of quarantined waste (urine, faeces, dirty linen, etc.) [88,89].

### 3.3. Decontamination

It should be emphasised that there is a lack of suggestions available about decontamination procedures due to the Novichok poisoning. However, it should be mentioned that the Treaty Manual of the Organisation for the Prevention of Chemical Weapons (OPCW) draws on previous human poisonings and the recent experience of sarin poisoning in Syria [90]. They stressed the importance of decontamination to prevent continued patient poisoning and continued patient poisoning protect other patients and personnel close to poisoned patients [91]. The type and level of decontamination depend on the physical properties of the Novichoks, if any, and the exposure route. Liquid Novichoks can be absorbed or evaporated through the skin, causing a danger of inhalation to those around them). On the contrary, Novichoks will be potentially systemically absorbed, metabolised or naturally expelled from the patient’s respiratory system. Removing clothing (distinction) will provide at least 80% of the decontamination because the clothing fibre can capture and hold liquid Novichoks and steam. After decoupling, liquid agents must also be cleaned with materials such as Fuller’s Earth (a fine adsorbent powder used by the army) or clinical paper towels (blue rolls) and then washed with soapy water at 35 °C.

## 4. Conclusions

Novichoks are the subject of considerable interest nowadays, although many unclear issues still need clarification. A full explanation of their structure and properties is necessary to better understand the threats these compounds pose and develop adequate protection against them. The potentially dangerous nature of the Novichoks used as chemical weapons poses a serious and global threat to the lives of casualties. The incidents in Salisbury and Amesbury only confirmed their extreme toxicity and the need to develop effective procedures to organise a medical response. In addition, introducing solutions to protect medical personnel when in contact with a hazardous substance increases the chances of saving lives. Therefore, the threat posed by Novichoks needs to be urgently assessed in order to be able to deal with future terrorist attacks or their use as chemical weapons. Developing efficient and effective HAZMAT/CBRNE approaches in contact with threatening A-series nerve agents is crucial. Despite the number of poisonings with these OPs being negligible, physicians ought to be familiar with the management to protect the life of poisoning victims. We hope that this review will stimulate future research and help fill in missing data gaps, accelerate progress in improving our protection, and help develop an optimal treatment for casualties of Novichok poisoning.

## Data Availability

All the data are available for this manuscript.
